# Oral health in patients with inflammatory bowel disease: A cross-sectional survey in Sweden

**DOI:** 10.1007/s00784-024-05951-5

**Published:** 2024-10-05

**Authors:** Kristina Bertl, Johan Burisch, Nikolaos Pandis, Björn Klinge, Andreas Stavropoulos

**Affiliations:** 1 Department of Periodontology, Dental Clinic, Faculty of Medicine, Sigmund Freud University Vienna, Vienna, Austria; 2 Department of Periodontology, Blekinge Hospital, Karlskrona, Sweden; 3 Gastrounit, Medical Division, Copenhagen University Hospital - Amager and Hvidovre, Hvidovre, Denmark; 4 Copenhagen Center for Inflammatory Bowel Disease in Children, Adolescents and Adults, Copenhagen University Hospital - Amager and Hvidovre, Hvidovre, Denmark; 5 Department of Orthodontics and Dentofacial Orthopedics, School of Dental Medicine, University of Bern, Bern, Switzerland; 6 Periodontology, Faculty of Odontology, University of Malmö, Malmö, Sweden; 7 Department of Dental Medicine, Division of Oral Health and Periodontology, Karolinska Institute, Stockholm, Sweden; 8 Division of Conservative Dentistry and Periodontology, University Clinic of Dentistry, Medical University of Vienna, Vienna, Austria; 9 Department of Periodontology, School of Dental Medicine, University of Bern, Bern, Switzerland

**Keywords:** Crohn’s disease, Inflammatory bowel disease, Oral health, Survey, Ulcerative colitis

## Abstract

**Objectives:**

The aim of this cross-sectional survey was to assess oral health, including prevalence of periodontitis and rate of tooth loss, in a Swedish cohort of patients with inflammatory bowel disease (IBD).

**Methods:**

A questionnaire on general anamnestic and socio-economic aspects, IBD diagnosis, and various oral health aspects was distributed online. The analyses focused on the comparison between patients diagnosed with ulcerative colitis (UC) or Crohn’s disease (CD) as well as on factors associated with self-reported severe periodontitis and tooth loss.

**Results:**

Analyses were based on answers from 786 patients; 415 with UC, 371 with CD, 74% female. In both disease entities, high prevalence of severe periodontitis (i.e., 38.5%) was reported, and about 19% of the population had less than 20 remaining teeth and 6.5% a poor oral health-related quality of life. CD patients tended to be more severely affected than UC patients (*p* > 0.05 in the adjusted analysis). Almost 90% of CD patients were aware of being entitled to a bi-annual governmental financial support for dental care due to IBD; however, 1 out of 4 UC patients did not. Furthermore, IBD patients largely believe that the interest of their physicians in any oral lesions due to IBD diagnosis is low.

**Conclusions:**

Severe periodontitis and high rate of tooth loss are frequent in Swedish IBD patients.

**Clinical relevance:**

Even though IBD patients receive bi-annually some special financial support for dental care, it seems this is still not sufficient and more preventive measures appear necessary.

## Introduction

Periodontitis has been linked to various systemic diseases, such as diabetes [1, 2], cardiovascular diseases [3], rheumatoid arthritis [4, 5], or chronic kidney disease [6]. More recently, there has been increased focus on the potential association between inflammatory bowel diseases (IBD), i.e., ulcerative colitis (UC) and Crohn’s disease (CD), and periodontal diseases. This is evident by the high number of recent preclinical and clinical trials, and the high number of (systematic) reviews on this topic [for overview see: [7–14]]. This association might – at least partly – be explained by the rather comparable etiopathogenesis of both diseases. Specifically, in both diseases the immune system of a susceptible host reacts with an (excessive) inflammatory response on a bacterial trigger/dysbiotic biofilm, which in turn initiates tissue damage either in the bowel or at the tooth supporting structures [15–20]. In addition, accumulating evidence indicates that bacterial transition between the oral cavity and the bowel might play another important role [21]. For example, it has been reported that specific oral bacteria such as *Porphyromonas gingivalis* or *Fusobacterium nucleatum* potentially aggravate the severity and/or activity of IBD [22–24].

In this context, our group recently performed a large questionnaire-based, case–control study in Denmark, including about 1100 IBD patients and 3400 non-IBD individuals [25–28]. Specifically, IBD patients reported significantly worse oral health, and a higher prevalence of severe periodontitis and tooth loss compared to IBD-free controls [25]. In addition, IBD patients reported a significantly higher prevalence of problems in various daily live activities due to the state of their teeth or mouth and more frequent visits at the dentist, including receiving more dental treatment and spending more money compared to controls [26, 27]. In addition, these oral problems were associated with a higher IBD disability score, an increased IBD activity in the last 12 months, and an impaired IBD-specific health-related quality of life (QoL) [26, 28]. In general, CD patients tended to be more severely affected than UC patients, regarding several of the above-mentioned issues.

A recent Norwegian study, although based on a limited number of patients (i.e., 50 UC patients), did not confirm the results reported above for Denmark, i.e., the prevalence of periodontitis – although very high (i.e., 74%) – was comparable to a general Norwegian population and no correlation was found between periodontal parameters and UC disease indices [29]. Considering that, in general, there are similarities in genetic background, lifestyle, and health-care systems among the Scandinavian countries, the discrepancy in the findings between the Norwegian and Danish studies is somehow surprising. In this context, there is only scarce information about oral health in IBD patients in Sweden [30], and the prevalence of periodontitis and extend of edentulism in this group of patients is unknown. Hence, the aim of the present cross-sectional survey was to assess oral health aspects including amongst others periodontal health, tooth loss rate, oral lesions, and patient reported outcomes in a Swedish cohort of IBD patients and to compare the data provided by patients with either UC or CD, using the online questionnaire previously distributed in Denmark [25].

## Materials and methods

### Distribution of the questionnaire

The present cross-sectional study was performed in Sweden as an online survey. The content of the questionnaire was identical to the content of the questionnaire previously distributed to Danish IBD patients [25–28], but translated in Swedish, and was distributed to members of the Swedish Patient Organisation for Gastrointestinal Diseases (2300 members with IBD; https://magotarm.se/). Responses were collected via a web-based survey tool (Sunet Survey) for three months from February to May 2022; within this period, two reminders were sent. All data were collected anonymously, and an ethical approval was not required by the local authorities.

### Content of the questionnaire

The questionnaire addressed general anamnestic and socio-economic aspects, IBD- and oral health-related characteristics. The results are reported in two manuscripts: the current one is focusing on oral health-related aspects and another one focusing on IBD-related aspects [31]. Hence, the following parts of the survey have been used for the analysis herein: gender, age, body height and weight for calculation of the body mass index (BMI), comorbidities, smoking status, living area, education, income after taxes, IBD diagnosis [UC, CD, or unclassified IBD (IBD-U)], and oral health-related characteristics. Regarding the oral health-related characteristics, the following information was collected: number of remaining teeth, self-perceived state of teeth and gums, presence of a tooth replacement, details on regular oral hygiene measures, reasons for- and time passed since last visit for professional dental care, money spent for professional dental care in the last 12 months, awareness of being entitled, within the public health insurance, to a bi-annual special financial support for dental care (STB; “särskilt tandvårdsbidrag”) due to IBD, details on oral lesions, oral health-related QoL aspects, and questions previously recommended for self-reported surveillance of periodontitis [32]. The questions on oral health-related QoL have been partly taken from the World Health Organization (WHO) Oral Health Questionnaire (for details see Table 3) [33], and included the 5 questions of the oral health impact profile (OHIP)-5. These questions cover functional limitation, pain, psychological discomfort, physical disability, and handicap [34, 35]. The total sum (ranging from 0 to 20) as well as the following dichotomous discrimination between good and poor oral health-related QoL was calculated: if up to one oral impact was experienced “fairly often” or “very often”, the participant was judged as having a good oral health-related QoL, while 2 or more oral impacts reported “fairly often” or “very often” indicated a poor oral health-related QoL [35]. Finally, based on 5 questions for self-reported surveillance of periodontitis, age, and smoking status, the Periodontal Screening Score (PESS) [36] was calculated. A PESS ≥ 5 has been previously validated for identifying severe periodontitis cases [36].

### Statistical analysis

Frequency distribution for categorical variables and mean (standard deviation) or median (interquartile ranges) for continuous variables were reported for all IBD patients, as well as separately for patients with UC or CD. Patients reporting to have IBD-U were included in the group of UC patients. To assess any differences between UC and CD, Fisher’s exact test or chi-squared tests were used for categorical parameters (i.e., chi-squared test was used if each cell presented with a frequency greater than 5, otherwise Fisher’s exact test was used). For continuous variables, either the independent *t*-test (for normally distributed data) or the Mann–Whitney U test (for data lacking normal distribution) was used. Distribution of data was assessed graphically using Q-Q-plots and the Shapiro–Wilk test.

Two primary dichotomous outcome parameters were chosen, which were assessed using binary logistic regression models: 1) PESS (score < 5 versus ≥ 5), and 2) tooth number (≥ 20 teeth versus < 20 teeth). Patient group (UC / CD) was defined as the main predictor and age, gender, and smoking status (never / former / current) as a priori confounders. Further, the following additional confounders were considered: 1) diabetes (yes / no); 2) osteoporosis (yes / no); 3) rheumatoid arthritis (yes / no); 4) ankylosing spondylitis (yes / no); 5) psoriasis (yes / no); 6) depression (yes / no); 7) high cholesterol (yes / no); 8) cardiovascular disease (yes / no); 9) asthma (yes / no); 10) chronic obstructive pulmonary disease (COPD; yes / no); 11) living area (city / suburban area / countryside); 12) education (no school or primary school / high school / higher education up to 3 years / higher education up to 6 years and / or PhD); 13) income after taxes [< 10,000 SEK (ca. 870 €) / 10,000 to < 20,000 SEK / 20,000 to < 30,000 SEK / ≥ 30,000 SEK]; and 14) BMI. In the analysis for PESS, “tooth number” (≥ 20 teeth / < 20 teeth) and in the analysis for tooth number, “PESS” (score < 5 / ≥ 5) was added as additional confounder. In a first step, the main predictor and each confounder were assessed in an univariable analysis, which was corrected for the a priori confounders (i.e., age, gender, and smoking status). In a second step, the main predictor, the a priori confounders, and all confounders presenting a p-value < 0.2 in the univariable analysis were combined in a multivariable analysis. Statistical analysis was performed with STATA/IC 17.0 for Mac and a p-value of ≤ 0.05 was considered as statistically significant.

## Results

### Response rate

Within 3 months, 786 IBD patients responded to the survey (response rate based on the known number of members with IBD: 34.2%); 368 patients reported to be diagnosed with UC, 371 with CD, and 47 with IBD-U.

### General characteristics

The general characteristics of all patients and separately for patients with UC and CD are displayed in Table 1. About 74% of the patients were female and, in general, patients with UC and CD were well comparable. However, a few statistically significant differences were observed between UC and CD patients. More specifically, patients with CD were slightly older (57.9 versus 55.2 years, respectively; p = 0.015) and more frequently diagnosed with osteoporosis (12.1 versus 7.2%, respectively; p = 0.020) and ankylosing spondylitis (4.6 versus 1.7%, respectively; p = 0.019), but had less often a higher education up to 6 years (with or without a PhD) (18.6 versus 31.3%, respectively; p < 0.001) and an income ≥ 20,000 SEK (ca. 1740 €) (41.2 versus 51.6%, respectively; p = 0.007) compared to patients with UC.

**Table 1:** Self-reported general characteristics of all IBD patients (*n* = 786) and separately for patients with ulcerative colitis (including unclassified inflammatory bowel disease; *n* = 415) or Crohn’s disease (n = 371)

**Parameter**		**All IBD patients**	**Ulcerative colitis**	**Crohn’s disease**	***p*-value**^1^
Gender [n (%)]	*Female*	580 (73.8)	307 (74.0)	273 (73.6)	0.901
	*Male*	206 (26.2)	108 (26.0)	98 (26.4)	
Age [mean (S.D.)]		56.5 (15.4)	55.2 (15.2)	57.9 (15.5)	**0.015**
BMI [median (Q1; Q3)]		24.8 (22.2; 28.0)	24.8 (22.4; 28.2)	24.8 (21.9; 27.7)	0.296^2^
Comorbidities [yes; n (%)]	*Diabetes*	37 (4.7)	21 (5.1)	16 (4.3)	0.621
	*Osteoporosis*	75 (9.5)	30 (7.2)	45 (12.1)	**0.020**
	*Rheumatoid arthritis*	35 (4.5)	15 (3.6)	20 (5.4)	0.228
	*Ankylosing spondylitis*	24 (3.1)	7 (1.7)	17 (4.6)	**0.019**
	*Psoriasis*	51 (6.5)	23 (5.5)	28 (7.6)	0.255
	*Depression*	73 (9.3)	39 (9.4)	34 (9.2)	0.910
	*High cholesterol*	47 (6.0)	28 (6.8)	19 (5.1)	0.337
	*Cardiovascular disease*	173 (22.0)	81 (19.5)	92 (24.8)	0.074
	*Asthma*	85 (10.8)	45 (10.8)	40 (10.8)	0.978
	*COPD*	12 (1.5)	5 (1.2)	7 (1.9)	0.563^3^
Smoking [n (%)]	*Never*	361 (45.9)	202 (48.7)	159 (42.9)	0.155
	*Former*	332 (42.3)	162 (39.0)	170 (45.8)	
	*Current*	93 (11.8)	51 (12.3)	42 (11.3)	
Living area [n (%)]	*City*	415 (52.8)	220 (53.0)	195 (52.6)	0.992
	*Suburban area*	240 (30.5)	126 (30.4)	114 (30.7)	
	*Countryside*	131 (16.7)	69 (16.6)	62 (16.7)	
Education [n (%)]	*No school*	1 (0.1)	0 (0)	1 (0.3)	** < 0.001**^**3**^
	*Primary school*	63 (8.0)	24 (5.8)	39 (10.5)	
	*High school*	281 (35.8)	137 (33.0)	144 (38.8)	
	*Higher education up to 3 years*	242 (30.8)	124 (29.9)	118 (31.8)	
	*Higher education up to 6 years*	186 (23.7)	120 (28.9)	66 (17.8)	
	*PhD*	13 (1.6)	10 (2.4)	3 (0.8)	
Income [n (%)]	< *10,000 SEK*	81 (10.3)	31 (7.5)	50 (13.5)	**0.007**
	*10,000 to* < *20,000 SEK*	338 (43.0)	170 (40.9)	168 (45.3)	
	*20,000 to* < *30,000 SEK*	247 (31.4)	144 (34.7)	103 (27.7)	
	≥ *30,000 SEK*	120 (15.3)	70 (16.9)	50 (13.5)	

^1^Chi-squared test for categorical variables and independent t-test for continuous variable was applied unless indicated otherwise

^2^Mann–Whitney U-test was applied

^3^Fisher’s exact test was applied

Bold values indicate significance.

*BMI* body mass index, *IBD* inflammatory bowel disease, *Q1/Q3* first/third quartile, *S.D.* standard deviation, *SEK* Swedish krone

### Dental and periodontal characteristics

The self-reported dental and periodontal characteristics of all patients and separately for patients with UC and CD are displayed in Table 2. Patients with CD appeared to have a higher prevalence of periodontal problems compared to patients with UC. More specifically, significantly more CD patients had less than 20 remaining teeth (24.0 versus 14.7%, respectively; p = 0.009) and self-reported severe periodontitis (43.0 versus 34.5%, respectively; p = 0.014) compared to patients with UC. Furthermore, significantly more CD patients used interdental brushes (58.2 versus 48.9%, respectively; p = 0.009) and spent ≥ 3000 SEK (ca. 260 €) for professional dental care in the last 12 months (28.7 versus 18.9%, respectively; p = 0.024), but CD patients were also more often aware of being entitle for STB (89.2 versus 73.7%, respectively; p < 0.001) compared to UC patients.

**Table 2:** Self-reported dental and periodontal characteristics and dental care habits of all IBD patients (*n* = 786) and separately for patients with ulcerative colitis (including unclassified inflammatory bowel disease; *n* = 415) or Crohn’s disease (*n* = 371)

**Parameter**		**All IBD patients**	**Ulcerative colitis**	**Crohn’s disease**	***p*-value**^1^
Tooth number [n(%)]	*Edentulous*	8 (1.0)	4 (1.0)	4 (1.1)	**0.009**^2^
	*1 to 9 teeth*	31 (4.0)	12 (2.9)	19 (5.1)	
	*10 to 19 teeth*	111 (14.1)	45 (10.8)	66 (17.8)	
	≥ *20 teeth*	636 (80.9)	354 (85.3)	282 (76.0)	
PESS ≥ 5 [yes; n (%)]^3^	*All patients*	302 (38.5)	143 (34.5)	159 (43.0)	**0.014**
	< *40 years*	2 (1.6)	0 (0.0)	2 (3.7)	
	*40 to 54 years*	31 (15.7)	12 (10.6)	19 (22.4)	
	≥ *55 years*	269 (58.2)	131 (56.7)	138 (59.7)	
PESS [n (%)]^4^	*1 to 4*	482 (61.6)	272 (65.9)	210 (56.9)	**0.036**
	*5 to 8*	265 (33.9)	124 (30.0)	141 (38.2)	
	*9 to 13*	35 (4.5)	17 (4.1)	18 (4.9)	
Overall, how would you rate the health of your teeth and gums? (n [%])	*Excellent*	44 (5.6)	22 (5.3)	22 (5.9)	0.066^2^
	*Very good*	177 (22.5)	107 (25.8)	70 (18.9)	
	*Good*	268 (34.1)	147 (35.4)	121 (32.6)	
	*Fair*	206 (26.2)	100 (24.1)	106 (28.6)	
	*Poor*	87 (11.1)	37 (8.9)	50 (13.5)	
	*Do not know*	4 (0.5)	2 (0.5)	2 (0.5)	
Tooth replacement^5^ [present; n (%)]	*Partial denture*	26 (3.3)	11 (2.7)	15 (4.0)	0.276
	*Full upper denture*	14 (1.8)	5 (1.2)	9 (2.4)	0.280^2^
	*Full lower denture*	6 (0.8)	3 (0.7)	3 (0.8)	1.000^2^
	*Dental implants*	156 (19.9)	73 (17.6)	83 (22.4)	0.093
Frequency of toothbrushing [n(%)]	*Never*	1 (0.1)	0 (0)	1 (0.3)	0.164^2^
	*Once a month*	1 (0.1)	0 (0)	1 (0.3)	
	*2- to 3-times a month*	1 (0.1)	1 (0.2)	0 (0)	
	*Once a week*	1 (0.1)	1 (0.2)	0 (0)	
	*2- to 6-times a week*	3 (0.4)	0 (0)	3 (0.8)	
	*Once a day*	77 (9.8)	38 (9.2)	39 (10.5)	
	*Twice or more a day*	702 (89.4)	375 (90.4)	327 (88.1)	
Oral hygiene measures^5^ [used; n (%)]	*Interdental brush*	419 (53.3)	203 (48.9)	216 (58.2)	**0.009**
	*Thread (dental floss)*	500 (63.6)	273 (65.8)	227 (61.2)	0.181
	*Wooden toothpick*	129 (16.4)	65 (15.7)	64 (17.3)	0.548
	*Plastic toothpick*	394 (50.1)	199 (48.0)	195 (52.6)	0.197
	*Charcoal*	1 (0.1)	1 (0.2)	0 (0)	1.000^2^
	*Chewstick/Miswak*	1 (0.1)	0 (0)	1 (0.3)	0.472^2^
	*Toothpaste*	783 (99.6)	415 (100)	368 (99.2)	0.105^2^
Last dental visit [n (%)]	*Never*	0 (0)	0 (0)	0 (0)	0.406^2^
	≥ *5 years ago*	15 (1.9)	6 (1.4)	9 (2.4)	
	*2 to 5 years ago*	52 (6.6)	26 (6.3)	26 (7.0)	
	*1 to 2 years ago*	68 (8.7)	39 (9.4)	29 (7.8)	
	*6 to 12 months ago*	186 (23.6)	107 (25.8)	79 (21.3)	
	≤ *6 months ago*	465 (59.2)	237 (57.1)	228 (61.5)	
Reason for last dental visit [n (%)]	*Consultation*	6 (0.8)	2 (0.5)	4 (1.1)	0.509^2^
	*Pain and/or oral health problems*	95 (12.1)	45 (10.8)	50 (13.5)	
	*Treatment*	133 (16.9)	67 (16.1)	66 (17.8)	
	*Regular check-up*	546 (69.4)	297 (71.6)	249 (67.1)	
	*Don’t know*	6 (0.8)	4 (1.0)	2 (0.5)	
Money spent for professional dental care in the last 12 months [n (%)]^6^	< *1500 SEK*	306 (47.0)	174 (50.6)	132 (43.0)	**0.024**
	*1500 to* < *3000 SEK*	192 (29.5)	105 (30.5)	87 (28.3)	
	*3000 to* < *9000 SEK*	115 (17.7)	51 (14.8)	64 (20.9)	
	≥ *9000 SEK*	38 (5.8)	14 (4.1)	24 (7.8)	
**Awareness of STB** [yes; n (%)]		637 (81)	306 (73.7)	331 (89.2)	** < 0.001**

^1^Chi-squared test was applied unless indicated otherwise

^2^Fisher’s exact test was applied

^3^Based on 785 responses

^4^Based on 782 responses

^5^Multiple answers possible

^6^Based on 651 participants, who have been with the dentist in the last 12 months

Bold values indicate significance.

*IBD* inflammatory bowel disease, *PESS* periodontal screening score, *SEK* Swedish krone, *STB* “särskilda tandvårdsbidraget” (special financial support for dental care due to IBD within the public health insurance)

### Oral-health related QoL aspects

The self-reported oral-health related QoL aspects of all patients and separately for patients with UC and CD are displayed in Table 3. Patients with CD reported for 6 out of 12 questions of the WHO Oral Health Questionnaire a significantly higher prevalence of problems in various daily live activities due to the state of their teeth or mouth in the last 12 months compared to UC patients. Among the questions with a significant difference, CD patients answered about 1.3- to 4.2-times more often with “fairly often” or “very often” compared to UC patients. In addition, in 3 out of 5 questions of the OHIP-5 questionnaire a significant difference was detected between UC and CD patients. Among the questions with a significant difference, CD patients answered about 1.4- to 8.6-times more often with “fairly often” or “very often” compared to UC patients. Overall, the prevalence of an answer “fairly often” or “very often”, ranged for UC patients between 0.5 and 5.8%, while for CD patients between 4.3 and 8.6%. Thus, CD patients had a significantly higher prevalence of poor oral health-related QoL (9.2% versus 4.1%, respectively; *p* = 0.004) and a significantly higher OHIP-5 score (*p* = 0.001) compared to UC patients.

**Table 3:** Self-reported oral-health related quality of life aspects of all IBD patients (*n* = 786) and separately for patients with ulcerative colitis (including unclassified inflammatory bowel disease; *n* = 415) or Crohn’s disease (*n* = 371)

**Parameter**			**All IBD patients**	**Ulcerative colitis**	**Crohn’s disease**	***p*-value**^1^
Because of the state of your teeth or mouth, how often have you experienced any of the following problems during the past 12 months?^3^	**Difficulty in biting foods** [n (%)]	*Very often*	18 (2.3)	7 (1.7)	11 (3.0)	**0.024**^2^
		*Fairly often*	51 (6.5)	20 (4.8)	31 (8.4)	
		*Sometimes*	202 (25.7)	99 (23.8)	103 (27.8)	
		*No*	503 (64.0)	285 (68.7)	218 (58.7)	
		*Don’t know*	12 (1.5)	4 (1.0)	8 (2.1)	
	**Difficulty chewing foods** [n (%)]	*Very often*	14 (1.8)	4 (1.0)	10 (2.7)	**0.006**^2^
		*Fairly often*	39 (5.0)	15 (3.6)	24 (6.5)	
		*Sometimes*	181 (23.0)	84 (20.2)	97 (26.1)	
		*No*	546 (69.4)	310 (74.7)	236 (63.6)	
		*Don’t know*	6 (0.8)	2 (0.5)	4 (1.1)	
	**Difficulty with speech/trouble pronouncing words** [n (%)]	*Very often*	3 (0.4)	0 (0)	3 (0.8)	0.061^2^
		*Fairly often*	15 (1.9)	5 (1.2)	10 (2.7)	
		*Sometimes*	94 (12.0)	50 (12.1)	44 (11.8)	
		*No*	658 (83.7)	355 (85.5)	303 (81.7)	
		*Don’t know*	16 (2.0)	5 (1.2)	11 (3.0)	
	**Dry mouth** [n (%)]	*Very often*	133 (16.9)	57 (13.7)	76 (20.5)	**0.009**^2^
		*Fairly often*	137 (17.5)	68 (16.4)	69 (18.6)	
		*Sometimes*	269 (34.2)	139 (33.5)	130 (35.0)	
		*No*	236 (30.0)	143 (34.5)	93 (25.1)	
		*Don’t know*	11 (1.4)	8 (1.9)	3 (0.8)	
	**Felt embarrassed due to appearance of teeth** [n (%)]	*Very often*	37 (4.7)	13 (3.1)	24 (6.5)	**0.010**
		*Fairly often*	49 (6.2)	18 (4.3)	31 (8.3)	
		*Sometimes*	200 (25.5)	101 (24.3)	99 (26.7)	
		*No*	488 (62.1)	277 (66.8)	211 (56.9)	
		*Don’t know*	12 (1.5)	6 (1.5)	6 (1.6)	
	**Felt tense because of problems with teeth or mouth **[n (%)]	*Very often*	42 (5.4)	21 (5.1)	21 (5.7)	**0.004**
		*Fairly often*	63 (8.0)	27 (6.5)	36 (9.7)	
		*Sometimes*	203 (25.8)	89 (21.5)	114 (30.7)	
		*No*	456 (58.0)	267 (64.3)	189 (50.9)	
		*Don’t know*	22 (2.8)	11 (2.6)	11 (3.0)	
	**Have avoided smiling because of teeth** [n (%)]	*Very often*	28 (3.6)	12 (2.9)	16 (4.3)	0.106
		*Fairly often*	37 (4.7)	17 (4.1)	20 (5.4)	
		*Sometimes*	133 (16.9)	59 (14.2)	74 (20.0)	
		*No*	576 (73.3)	321 (77.3)	255 (68.7)	
		*Don’t know*	12 (1.5)	6 (1.5)	6 (1.6)	
	**Had sleep that is often interrupted** [n (%)]	*Very often*	47 (6.0)	21 (5.1)	26 (7.0)	0.446
		*Fairly often*	83 (10.5)	45 (10.8)	38 (10.2)	
		*Sometimes*	177 (22.5)	88 (21.2)	89 (24.0)	
		*No*	440 (56.0)	243 (58.6)	197 (53.1)	
		*Don’t know*	39 (5.0)	18 (4.3)	21 (5.7)	
	**Have taken days off work** [n[n (%)]	*Very often*	4 (0.5)	1 (0.2)	3 (0.8)	** < 0.001**^2^
		*Fairly often*	24 (3.1)	5 (1.2)	19 (5.1)	
		*Sometimes*	78 (9.9)	47 (11.3)	31 (8.4)	
		*No*	626 (79.6)	343 (82.7)	283 (76.3)	
		*Don’t know*	54 (6.9)	19 (4.6)	35 (9.4)	
	**Difficulty doing usual activities** [n (%)]	*Very often*	7 (0.9)	1 (0.2)	6 (1.6)	0.099^2^
		*Fairly often*	26 (3.3)	11 (2.7)	15 (4.0)	
		*Sometimes*	111 (14.1)	57 (13.7)	54 (14.6)	
		*No*	621 (79.0)	338 (81.5)	283 (76.3)	
		*Don’t know*	21 (2.7)	8 (1.9)	13 (3.5)	
	**Felt less tolerant of spouse or**	*Very often*	7 (0.9)	4 (1.0)	3 (0.8)	0.132^2^
	**people who are close to you** [n	*Fairly often*	22 (2.8)	8 (1.9)	14 (3.8)	
	(%)]	*Sometimes*	144 (18.3)	67 (16.1)	77 (20.8)	
		*No*	572 (72.8)	317 (76.4)	255 (68.7)	
		*Don’t know*	41 (5.2)	19 (4.6)	22 (5.9)	
	**Have reduced participation**	*Very often*	18 (2.3)	7 (1.7)	11 (3.0)	0.062
	**in social activities** [n (%)]	*Fairly often*	40 (5.1)	15 (3.6)	25 (6.7)	
		*Sometimes*	101 (12.9)	57 (13.7)	44 (11.9)	
		*No*	600 (76.3)	326 (78.6)	274 (73.8)	
		*Don’t know*	27 (3.4)	10 (2.4)	17 (4.6)	
Within the last 7 days…	**...have you had difficulty chewing any food because of problems with your teeth, mouth, dentures or jaws?**	*Very often*	13 (1.7)	5 (1.2)	8 (2.2)	**0.037**^2^
		*Fairly often*	34 (4.3)	16 (3.9)	18 (4.9)	
		*Occasionally*	121 (15.5)	55 (13.3)	66 (17.9)	
		*Hardly ever*	147 (18.8)	69 (16.7)	78 (21.1)	
		*Never*	467 (59.7)	268 (64.9)	199 (53.9)	
	**…have you had painful aching in your mouth?**	*Very often*	15 (1.9)	5 (1.2)	10 (2.7)	0.079^2^
		*Fairly often*	38 (4.9)	19 (4.6)	19 (5.2)	
		*Occasionally*	156 (20.0)	77 (18.7)	79 (21.6)	
		*Hardly ever*	244 (31.4)	121 (29.4)	123 (33.6)	
		*Never*	325 (41.8)	190 (46.1)	135 (36.9)	
	**…have you felt uncomfortable about the appearance of your teeth or dentures?**	*Very often*	18 (2.3)	4 (1.0)	14 (3.9)	**0.026**^2^
		*Fairly often*	30 (3.9)	13 (3.2)	17 (4.7)	
		*Occasionally*	82 (10.6)	38 (9.2)	44 (12.1)	
		*Hardly ever*	136 (17.5)	74 (17.9)	62 (17.1)	
		*Never*	510 (65.7)	284 (68.7)	226 (62.2)	
	**…have you felt that there has been less flavour in your food because of problems with your teeth, mouth, dentures or jaws?**	*Very often*	8 (1.1)	3 (0.8)	5 (1.4)	0.525^2^
		*Fairly often*	21 (2.8)	9 (2.3)	12 (3.4)	
		*Occasionally*	116 (15.4)	59 (14.7)	57 (16.1)	
		*Hardly ever*	132 (17.5)	66 (16.5)	66 (18.6)	
		*Never*	477 (63.2)	263 (65.7)	214 (60.5)	
	**…have you had difficulty doing your usual jobs because of problems with your teeth, mouth, dentures or jaws?**	*Very often*	6 (0.8)	0 (0)	6 (1.6)	**0.001**^2^
		*Fairly often*	12 (1.5)	2 (0.5)	10 (2.7)	
		*Occasionally*	50 (6.4)	19 (4.6)	31 (8.5)	
		*Hardly ever*	92 (11.8)	50 (12.1)	42 (11.4)	
		*Never*	619 (79.5)	341 (82.8)	278 (75.8)	
OHIP judgement [n (%)]		*Poor*	51 (6.5)	17 (4.1)	34 (9.2)	**0.004**
OHIP sum		*Median (Q1; Q3)*	2 (0; 5)	2 (0; 4)	2 (1; 6)	**0.001**

^1^Chi-squared test for categorical variables and Mann Whitney-U test for continuous variable was applied unless indicated otherwise

^2^Fisher’s exact test was applied

^3^Questions are derived from the World Health Organization (WHO) Oral Health Questionnaire

Bold values indicate significance.

*IBD* inflammatory bowel disease, *OHIP* oral health impact profile, *Q1/Q3* first/third quartile

### Experience with oral lesions

The self-reported experience with oral lesions of all patients and separately for patients with UC and CD are displayed in Table 4. About 30% of the patients indicated having had at some timepoint problems with oral lesions without any significant difference between CD and UC patients; however, CD patients tended to have them more frequently in a generalized form compared to UC patients (39.7 versus 30.1%, respectively; p = 0.120). Both UC and CD patients judged the occurrence of oral lesions as unrelated to IBD activity in about 2/3 of the cases. Almost half of the patients judged the oral lesions to be from “average painful” to “very painful”, but only in about 25% of the cases the oral lesions affected “often”, “very often”, or “always” food intake and in ≤ 10% of the cases any social activities. Only 6.7% of the IBD patients, who experienced at some timepoint problems with oral lesions, indicated having received treatment for them; treatment was in about 56 and 25% of the cases delivered by the dentist or gastroenterologist, respectively. In general, there have been no differences between UC and CD patients regarding the above-mentioned parameters. Significant differences were only detected regarding the questions about whether a physician informed them about the connection between IBD and oral lesions (23.2 versus 10.8% of the CD and UC patients, respectively, answered with yes; *p* < 0.001) and about their opinion whether their physician is interested in problems with their mouth (21.8 versus 12.0% of the CD and UC patients, respectively, answered with yes; *p* = 0.001). Only about 15% of the patients believed that their physician knows how to treat oral lesions, i.e., 12.8 and 18.3% of the UC and CD patients, respectively (*p* = 0.098).

**Table 4:** Self-reported experience with oral lesions of all IBD patients (*n* = 786) and separately for patients with ulcerative colitis (including unclassified inflammatory bowel disease; *n* = 415) or Crohn’s disease (*n* = 371)

**Parameter**		**All IBD patients**	**Ulcerative colitis**	**Crohn’s disease**	*p*-**value**^1^
Has your physician informed you about the connection between IBD & oral lesions? [n (%)]	*Yes*	131 (16.7)	45 (10.8)	86 (23.2)	** < 0.001**
	*No*	587 (74.7)	340 (81.9)	247 (66.6)	
	*Don’t know*	68 (8.6)	30 (7.3)	38 (10.2)	
Do you think your physician is interested in problems with your mouth? [n (%)]	*Yes*	131 (16.7)	50 (12.0)	81 (21.8)	**0.001**
	*No*	266 (33.8)	153 (36.9)	113 (30.5)	
	*Don’t know*	389 (49.5)	212 (51.1)	177 (47.7)	
Do you think your physician knows how to treat oral lesions? [n (%)]	*Yes*	121 (15.4)	53 (12.8)	68 (18.3)	0.098
	*No*	203 (25.8)	111 (26.7)	92 (24.8)	
	*Don’t know*	462 (58.8)	251 (60.5)	211 (56.9)	
Do you regularly have oral lesions and if so, when was the last time you experienced them? [n (%)]	*Within the last week*	75 (9.5)	33 (8.0)	42 (11.3)	0.629
	*1 to 4 weeks ago*	61 (7.8)	36 (8.7)	25 (6.8)	
	*1 to 3 months ago*	30 (3.8)	15 (3.6)	15 (4.0)	
	*3 to 6 months ago*	34 (4.3)	18 (4.3)	16 (4.3)	
	≥ *7 months ago*	39 (5.0)	21 (5.1)	18 (4.9)	
	*No oral lesions*	547 (69.6)	292 (70.3)	255 (68.7)	
Are the oral lesions localized or generalized? [n (%)]^2^	*Localized*	156 (65.3)	86 (69.9)	70 (60.3)	0.120
	*Generalized*	83 (34.7)	37 (30.1)	46 (39.7)	
How do the oral lesions occur in relation to your IBD? [n (%)]^2^	*Simultaneously with the flare-up of the IBD*	45 (18.8)	20 (16.3)	25 (21.6)	0.197^3^
	*Before the flare-up of the IBD*	26 (10.9)	16 (13.0)	10 (8.6)	
	*After the flare-up of the IBD*	11 (4.6)	3 (2.4)	8 (6.9)	
	*In no relation to the IBD*	157 (65.7)	84 (68.3)	73 (62.9)	
How painful are the oral lesions? [n (%)]^2^	*Not painful at all*	16 (6.7)	6 (4.9)	10 (8.6)	0.604
	*Slightly painful*	111 (46.4)	60 (48.8)	51 (44.0)	
	*Average painful*	80 (33.5)	42 (34.1)	38 (32.7)	
	*Very painful*	32 (13.4)	15 (12.2)	17 (14.7)	
Are the oral lesions preventing you from eating what you want? [n (%)]^2^	*Always*	6 (2.5)	2 (1.6)	4 (3.5)	0.539^3^
	*Very often*	20 (8.4)	7 (5.7)	13 (11.2)	
	*Often*	28 (11.7)	14 (11.4)	14 (12.1)	
	*Sometimes*	90 (37.7)	48 (39.0)	42 (36.2)	
	*Seldom*	70 (29.3)	40 (32.5)	30 (25.8)	
	*Never*	25 (10.4)	12 (9.8)	13 (11.2)	
Do you feel that the oral lesions	*Always*	2 (0.9)	1 (0.8)	1 (0.9)	0.787
negatively affect your social	*Very often*	7 (2.9)	3 (2.4)	4 (3.4)	
activity, sports/leisure, personal relationships? [n (%)]^2^	*Often*	12 (5.0)	5 (4.1)	7 (6.0)	
	*Sometimes*	45 (18.8)	20 (16.3)	25 (21.6)	
	*Seldom*	73 (30.6)	38 (30.9)	35 (30.2)	
	*Never*	100 (41.8)	56 (45.5)	44 (37.9)	
Do you get any treatment for the oral lesions? [n (%)]^2^	*Yes*	16 (6.7)	7 (5.7)	9 (7.8)	0.523
If you get treatment for the oral lesions, who gave it to you? [n (%)]^4,5^	*Dentist*	9 (56.3)	6 (85.7)	3 (33.3)	0.060^3^
	*Gastroenterologist*	4 (25.0)	0 (0)	4 (44.4)	0.088^3^
	*General practitioner*	2 (12.5)	1 (14.3)	1 (11.1)	1.000^3^
	*Myself*	2 (12.5)	1 (14.3)	1 (11.1)	1.000^3^

^1^Chi-squared test was applied unless indicated otherwise

^2^Based on 239 IBD patients indicating having oral lesions

^3^Fisher’s exact test was applied

^4^Based on the 16 IBD patients receiving treatment

^5^Multiple answers possible

Bold values indicate significance

*IBD* inflammatory bowel disease

### PESS

The results of the uni- and multivariable analyses for the outcome parameter “PESS ≥ 5” are presented in Appendix 1 and Table 5. In the multivariable analysis, the main predictor (i.e., patient group) did not reveal a significant difference between UC and CD patients for the presence of self-reported severe periodontitis (OR: 1.17; 95% CI: 0.82, 1.65). Among the confounders, less than 20 remaining teeth (OR: 3.36; 95% CI: 2.14, 5.27), the presence of rheumatoid arthritis (OR: 3.64; 95% CI: 1.56, 8.51), a higher age (OR: 1.08; 95% CI: 1.06, 1.09), and current smoking (OR: 5.34; 95% CI: 3.02, 9.43) significantly increased the odds of self-reported severe periodontitis.

**Table 5:** Results of the multivariable binary logistic regression analysis for the dichotomous outcome parameter “PESS ≥ 5”. An odds ratio (OR) above one indicates higher odds for a PESS ≥ 5 (i.e., presence of self-reported severe periodontitis); the model has been corrected for age, gender, and smoking status

Parameter		OR	95% CI		*p*-value
			Lower	Upper	
Patient group	*Ulcerative colitis*	Ref			
	*Crohn’s disease*	1.167	0.826	1.649	0.381
Tooth number	≥ *20 teeth*	Ref			
	< *20 teeth*	**3.357**	**2.139**	**5.269**	** < 0.001**
Osteoporosis	*Absent*	Ref			
	*Present*	1.351	0.759	2.405	0.307
Rheumatoid arthritis	*Absent*	Ref			
	*Present*	**3.639**	**1.557**	**8.505**	**0.003**
Depression	*Absent*	Ref			
	*Present*	1.752	0.954	3.218	0.071
Income	< 10,000 SEK	Ref			
	10,000 to < 20,000 SEK	0.693	0.369	1.300	0.253
	20,000 to < 30,000 SEK	0.903	0.467	1.746	0.761
	≥ 30,000 SEK	0.857	0.406	1.809	0.685
Age	*Years*	**1.075**	**1.058**	**1.092**	** < 0.001**
Gender	*Male*	Ref			
	*Female*	0.781	0.523	1.168	0.229
Smoking	*Never*	Ref			
	*Former*	1.245	0.852	1.820	0.258
	*Current*	**5.340**	**3.023**	**9.434**	** < 0.001**

Bold values indicate significance.

*CI* confidence interval, *OR* odds ratio, *PESS* periodontal screening score, *SEK* Swedish crowns.

### Tooth number

The results of the uni- and multivariable analyses for the outcome parameter “tooth number” are presented in Appendix 2 and Table 6. In the multivariable analysis, the main predictor (i.e., patient group) did not reveal a significant difference between UC and CD patients in the odds for less than 20 remaining teeth (OR: 1.43; 95% CI: 0.95, 2.16). Among the confounders, self-reported severe periodontitis (OR: 3.62; 95% CI: 2.31, 5.66), the presence of COPD (OR: 4.67; 95% CI: 1.17, 18.66), a higher age (OR: 1.03; 95% CI: 1.01, 1.05), and former smoking (OR: 1.87; 95% CI: 1.17, 3.01) significantly increased the odds for less than 20 remaining teeth, while living at the countryside (OR: 0.50; 95% CI: 0.26, 0.96) and a higher income (20,000 to < 30,000 SEK: OR: 0.42; 95% CI: 0.20, 0.87; ≥ 30,000 SEK: OR: 0.29; 95% CI: 0.11, 0.76) significantly decreased the odds for less than 20 remaining teeth.

**Table 6:** Results of the multivariable binary logistic regression analysis for the dichotomous outcome parameter “tooth number”. An odds ratio (OR) above one indicates higher odds to have less than 20 teeth; the model has been corrected for age, gender, and smoking status

Parameter		OR	95% CI		*p*-value
			Lower	Upper	
Patient group	*Ulcerative colitis*	Ref			
	*Crohn’s disease*	1.427	0.945	2.155	0.091
PESS	< *5*	Ref			
	≥ *5*	**3.618**	**2.314**	**5.656**	** < 0.001**
Osteoporosis	*Absent*	Ref			
	*Present*	1.492	0.816	2.728	0.194
Psoriasis	*Absent*	Ref			
	*Present*	1.884	0.922	3.850	0.082
Depression	*Absent*	Ref			
	*Present*	1.145	0.564	2.324	0.709
COPD	*Absent*	Ref			
	*Present*	**4.665**	**1.166**	**18.662**	**0.029**
Living area	*City*	Ref			
	*Suburban area*	1.144	0.721	1.814	0.568
	*Countryside*	**0.500**	**0.260**	**0.962**	**0.038**
Education	*No school/Primary school*	Ref			
	*High school*	0.732	0.365	1.467	0.379
	*Higher education up to 3 years*	0.895	0.437	1.833	0.761
	*Higher education up to 6 years and/or PhD*	0.538	0.237	1.222	0.139
Income	< 10,000 SEK	Ref			
	10,000 to < 20,000 SEK	0.770	0.401	1.481	0.434
	20,000 to < 30,000 SEK	**0.416**	**0.198**	**0.874**	**0.021**
	≥ 30,000 SEK	**0.294**	**0.114**	**0.756**	**0.011**
Age	*Years*	**1.032**	**1.014**	**1.052**	** < 0.001**
Gender	*Male*	Ref			
	*Female*	0.841	0.525	1.348	0.472
Smoking	*Never*	Ref			
	*Former*	**1.873**	**1.167**	**3.007**	**0.009**
	*Current*	1.618	0.817	3.201	0.167

Bold values indicate significance

*CI* confidence interval, *COPD* chronic obstructive pulmonary disease, *OR* odds ratio, *PESS* periodontal screening score; SEK, Swedish crowns

## Discussion

The results of the present cross-sectional survey in a Swedish cohort of IBD patients showed a prevalence of self-reported severe periodontitis, defined as PESS ≥ 5, of about 39%, about 19% of the participants had < 20 teeth, and about 37% of the respondents rated the health of their teeth and gums as fair or poor. In comparison, in the Danish study mentioned earlier [25], about 32% and 16% of the IBD patients had a PESS ≥ 5 and < 20 teeth, respectively, and about 42% rated the health of their teeth and gums as fair or poor. It seems thus that the periodontal status of Swedish IBD patients is overall comparable with that of the of the Danish IBD patients.

Scandinavian countries are in general known to provide high healthcare standards and equal access for the citizens. However, the IBD development shows a different trend in Denmark and Sweden [37, 38]. Specifically, in Denmark the overall incidence of UC increased more than 4-times (from 6.2 to 27.2 per 100,000) and for CD tripled (from 5.1 to 15.6 per 100,000) from 1980 to 2017. In contrast, a similar report from Sweden covering the period 1990 to 2014 described an annual increase of the annual incidence of UC and CD of approximately 7% between 1990 and 2001, but a decrease by about 1 to 2% per year from 2002 onwards [39]. In addition, there might be some differences between the countries specifically for dental health care, as the public health insurance system in Sweden offers extensive coverage for dental care for both youngsters and adults, while in Denmark dental care for adults is largely private [40]. Indeed, superior periodontal health and lower tooth loss rate has been previously reported in the general population in Sweden compared to Denmark [41]. Thus, one would expect that Swedish IBD patients have a somehow better periodontal status comparing with the Danish. Indeed, the finding of a higher overall prevalence of PESS ≥ 5 in the Swedish population herein, compared with that observed in the Danish study can be explained by the age differences between the two populations. More specifically, the Swedish IBD patients were on average approximately 10 years older than the Danish patients (57 and 48 years, respectively) and the percentage of patients being ≥ 55 years of age was in the Swedish population almost double compared to the Danish population (59 and 33%, respectively). It is well known that higher age is associated with worse periodontal health and in fact, higher age, specifically an age ≥ 55 years, has a significant impact on the calculation of PESS (i.e., increased score), which was used herein as surrogate to “identify” periodontitis patients [36]. Furthermore, when comparing the same age groups, i.e., < 40, 40–54, and ≥ 55 years of age, the Swedish population showed a lower prevalence of PESS ≥ 5 compared to the Danish population for about 3.1, 8.1, and 6.5%, respectively. In perspective, the low frequency of using a partial or full denture (i.e., approximately 6%) and of participants with less than 10 remaining teeth (i.e., approximately 5%), confirms the overall high standard of dental care in Sweden. For example, in Germany in 2014 about 12% of the population aged between 65 and 74 years has been edentulous, and the average number of teeth of this age group was about 17 teeth [42]. The rate of edentulous patients of the present cohort was 1% in total, and 1.3 and 2.6% if considering only participants ≥ 50 and ≥ 65 years of age, respectively. Further, the rate of participants having ≥ 20 remaining teeth was 81% in total, and 76 and 66% if considering only participants ≥ 50 and ≥ 65 years of age, respectively.

CD patients had herein a trend for more oral problems compared to UC patients. For example, 43 versus 35% of CD and UC patients, respectively, had self-reported severe periodontitis, and about 24 versus 15% of CD and UC patients, respectively, had less than 20 remaining teeth. This, in turn, resulted in about two times more CD patients having poor oral health-related QoL (i.e., 9.2 versus 4.1%, respectively). These results are well in agreement with a previous Swedish survey reporting that CD patients perceive their oral health to be worse, and to have more mouth-related problems and a higher need for dental treatment compared to IBD-free controls [30]. Furthermore, the finding herein that CD patients have more oral health problems, including worse periodontal status and fewer teeth, compared to UC patients is also well in agreement with the results of the Danish study [25–27] and previous systematic reviews on the topic [11, 14]. In this context, the Swedish health insurance system recognizes that IBD patients have a higher risk for worse oral health compared to the general population. Thus, IBD diagnosis qualifies for special financial support for dental care (STB), providing an additional coverage of around 50 €, bi-annually, aiming for better prevention of oral problems. Almost 90% of the CD patients were aware of this extra support, while one out of 4 UC patients did not know about it. This finding together with a low number of IBD patients declaring that their physician has informed them about the possibility of oral lesions due to their IBD disease (i.e., 11 and 23% of the UC and CD patients, respectively) somehow indicates the lower priority given to oral health by the medical community. Indeed, a similar underrepresentation of oral lesions in any discussions with a physician was seen in the Danish study [27], and the latest European Crohn’s and Colitis Organization consensus on extra-intestinal manifestation of IBD included strikingly limited information about oral health problems [43].

Clearly, the present survey has some inevitable limitations, as it lacks any clinical dental and medical examination, and no non-IBD control group was recruited. In addition, a relatively high percentage of female IBD patients (ca. 75%) answered the survey, something that does not represent the gender distribution in Swedish IBD patients, previously described as relatively well balanced [38]. However, it has been discussed previously that women tend to answer more likely online surveys [44] and any models used herein have been corrected for gender to minimize any potential gender effect. In addition, the present survey represents only a single timepoint without any further follow-up. To gain more insight on the potential connection and interplay between periodontal disease and IBD, clinical studies with a long-term follow-up are required, such as for example a prospective, population-based inception cohort study currently ongoing in Denmark [45]. The combination of collecting IBD- and oral-health related parameters as well as microbiological data from the oral cavity and the gut in a cohort recruited at the time of IBD diagnosis will hopefully provide more insight into the connection.

In conclusion, the present cross-sectional survey confirmed that Swedish IBD patients suffer frequently from oral health issues. Even though the incidence of IBD has not been increasing recently in Sweden, still about one out of 40 Swedes will be diagnosed with IBD during their lifetime [39]. Thus, IBD patients and their oral co-morbidities pose a high burden to the health care system, and it appears relevant that increased focus should be put on preventive measures for maintaining oral health, in addition to the currently existing ones (i.e., the limited optional financial support mentioned above). Increasing awareness about the link between IBD and oral diseases among physicians may also increase the transfer of information to IBD patients regarding the importance of oral health and prevention.

## Data Availability

Data are available upon reasonable request.

## Appendix

Table7

**Table 7:** Results of the univariable binary logistic regression analyses for the dichotomous outcome parameter “PESS ≥ 5”. An odds ratio (OR) above one indicates higher odds for a PESS ≥ 5 (i.e., presence of self-reported severe periodontitis); all models have been corrected for age, gender, and smoking status

Parameter		OR	95% CI		*p*-value
			Lower	Upper	
Patient group	*Ulcerative colitis*	Ref			
	*Crohn’s disease*	1.287	0.925	1.792	0.135
Tooth number	≥ *20 teeth*	Ref			
	< *20 teeth*	**3.374**	**2.175**	**5.233**	** < 0.001**
Diabetes	*Absent*	Ref			
	*Present*	0.864	0.398	1.874	0.711
Osteoporosis	*Absent*	Ref			
	*Present*	**1.717**	**0.999**	**2.950**	**0.050**
Rheumatoid arthritis	*Absent*	Ref			
	*Present*	**3.605**	**1.571**	**8.273**	**0.002**
Ankylosing spondylitis	*Absent*	Ref			
	*Present*	1.554	0.640	3.772	0.330
Psoriasis	*Absent*	Ref			
	*Present*	1.202	0.625	2.313	0.582
Depression	*Absent*	Ref			
	*Present*	**2.098**	**1.176**	**3.745**	**0.012**
High cholesterol	*Absent*	Ref			
	*Present*	1.305	0.682	2.494	0.421
Cardiovascular disease	*Absent*	Ref			
	*Present*	1.267	0.856	1.875	0.236
Asthma	*Absent*	Ref			
	*Present*	1.092	0.652	1.829	0.739
COPD	*Absent*	Ref			
	*Present*	0.799	0.231	2.758	0.722
Living area	*City*	Ref			
	*Suburban area*	0.885	0.607	1.290	0.525
	*Countryside*	0.918	0.577	1.461	0.718
Education	*No school/Primary school*	Ref			
	*High school*	0.908	0.481	1.712	0.765
	*Higher education up to 3 years*	1.146	0.600	2.191	0.680
	*Higher education up to 6 years and/or PhD*	1.199	0.607	2.369	0.601
Income	< 10,000 SEK	Ref			
	10,000 to < 20,000 SEK	0.620	0.343	1.123	0.115
	20,000 to < 30,000 SEK	0.679	0.364	1.265	0.222
	≥ 30,000 SEK	0.620	0.306	1.256	0.184
BMI	*Score*	1.006	0.973	1.039	0.740
Age	*Years*	**1.080**	**1.064**	**1.095**	** < 0.001**
Gender	*Male*	Ref			
	*Female*	0.788	0.542	1.146	0.212
Smoking	*Never*	Ref			
	*Former*	1.349	0.941	1.933	0.103
	*Current*	**5.761**	**3.318**	**10.004**	** < 0.001**

Bold values indicate significance.

*BMI* body mass index, *CI* confidence interval, *COPD* chronic obstructive pulmonary disease, *OR* odds ratio, *PESS* periodontal screening score, *SEK* Swedish crowns

Table 8

**Table 8:** Results of the univariable binary logistic regression analyses for the dichotomous outcome parameter “tooth number”. An odds ratio (OR) above one indicates higher odds to have less than 20 teeth; all models have been corrected for age, gender, and smoking status

Parameter		OR	95% CI		*p*-value
			Lower	Upper	
Patient group	*Ulcerative colitis*	Ref			
	*Crohn’s disease*	**1.639**	**1.118**	**2.403**	**0.011**
PESS	< *5*	Ref			
	≥ *5*	**3.613**	**2.355**	**5.541**	** < 0.001**
Diabetes	*Absent*	Ref			
	*Present*	0.932	0.405	2.147	0.868
Osteoporosis	*Absent*	Ref			
	*Present*	**2.227**	**1.290**	**3.845**	**0.004**
Rheumatoid arthritis	*Absent*	Ref			
	*Present*	1.088	0.453	2.614	0.851
Ankylosing spondylitis	*Absent*	Ref			
	*Present*	0.658	0.187	2.310	0.513
Psoriasis	*Absent*	Ref			
	*Present*	**2.052**	**1.058**	**3.982**	**0.034**
Depression	*Absent*	Ref			
	*Present*	**1.895**	**1.005**	**3.573**	**0.048**
High cholesterol	*Absent*	Ref			
	*Present*	0.764	0.358	1.630	0.486
Cardiovascular disease	*Absent*	Ref			
	*Present*	1.070	0.696	1.645	0.759
Asthma	*Absent*	Ref			
	*Present*	1.082	0.595	1.967	0.796
COPD	*Absent*	Ref			
	*Present*	**4.216**	**1.208**	**14.714**	**0.024**
Living area	*City*	Ref			
	*Suburban area*	1.309	0.860	1.992	0.208
	*Countryside*	0.617	0.337	1.130	0.118
Education	*No school/Primary school*	Ref			
	*High school*	0.569	0.300	1.077	0.083
	*Higher education up to 3 years*	0.666	0.348	1.274	0.219
	*Higher education up to 6 years and/or PhD*	**0.359**	**0.172**	**0.747**	**0.006**
Income	< 10,000 SEK	Ref			
	10,000 to < 20,000 SEK	0.591	0.326	1.072	0.084
	20,000 to < 30,000 SEK	**0.336**	**0.172**	**0.655**	**0.001**
	≥ 30,000 SEK	**0.211**	**0.088**	**0.504**	** < 0.001**
BMI	*Score*	1.009	0.973	1.045	0.645
Age	*Years*	**1.058**	**1.041**	**1.074**	** < 0.001**
Gender	*Male*	Ref			
	*Female*	1.065	0.695	1.634	0.772
Smoking	*Never*	Ref			
	*Former*	**1.965**	**1.277**	**3.022**	**0.002**
	*Current*	**2.308**	**1.240**	**4.297**	**0.008**

Bold values indicate significance.

*BMI* body mass index, *CI* confidence interval, *COPD* chronic obstructive pulmonary disease, *OR* odds ratio, *PESS* periodontal screening score, *SEK* Swedish crowns
